# Psychological distress among Afghan refugees in Norway as a function of their integration

**DOI:** 10.3389/fpsyg.2023.1143681

**Published:** 2023-04-18

**Authors:** Dixie Brea Larios, David L. Sam, Gro Mjeldheim Sandal

**Affiliations:** ^1^Department of Psychosocial Science, University of Bergen, Bergen, Norway; ^2^Center for International Health, University of Bergen, Bergen, Norway

**Keywords:** Afghans, integration, acculturation, depression, anxiety, refugees, psychological integration

## Abstract

**Background:**

Often, refugees are susceptible to mental health problems due to adversities experienced before, during, and after the flight. Through a cross-sectional study, the present study examines the relationship between different aspects of integration and psychological distress among Afghans living in Norway.

**Methods:**

The participants were recruited through e-mail invitations, refugee-related organizations, and social media platforms. The participants (*N* = 114) answered questions about integration across multiple dimensions (psychological, social, navigational, economic, and linguistic) in line with the Immigration Policy Lab index (IPL -12/24). Hopkins symptoms checklist (HSCL-25) was used to assess psychological distress.

**Results:**

Based on hierarchical multiple regression analysis, both the psychological dimension (0.269 *p* < 0.01) and the navigational dimension (0.358 *p* < 0.05) of integration predicted psychological distress.

**Discussion/Conclusion:**

The results suggest that the psychological aspects of integration, such as being part of a community, having feelings of security, and a sense of belonging, are beneficial for the mental health and well-being of the Afghans in Norway and contribute further to other aspects of integration.

## 1. Introduction

Conflict, social unrest, war, and other extreme experiences can adversely affect refugees’ mental health and increase vulnerability to depression, anxiety, and post-traumatic stress disorder (PTSD) (e.g., Kirmayer et al., 2011; Henkelmann et al., 2020; Schlechter et al., 2020). Also, experiences after the flight or forced migration, such as lack of access to health services combined with substandard living conditions, unemployment, and language barriers, might increase the risk for mental health problems (Fazel et al., 2005; Lindert et al., 2009; Jakobsen et al., 2011; Kirmayer et al., 2011; Nickerson et al., 2017; Kartal et al., 2019). Because of these circumstances, refugees may experience reduced quality of life, affecting their well-being, and preventing them from integrating into the host country (Haj-Younes et al., 2020; Walther et al., 2020; Gagliardi et al., 2021). The lack of access to adequate healthcare, support from friends and family, and difficulty navigating a new country can all add to the mental health burden experienced during migration. Despite improvements in refugee groups’ mental health and well-being, the connection between pre- and post-migration determinants, mental health, and integration, is still poorly understood (e.g., Fjeld-Solberg et al., 2020; Haj-Younes et al., 2020; Opaas et al., 2020; Strømme et al., 2020). A deeper understanding of pre-migration determinants, such as mental health problems and integration outcomes, might be needed. The perspectives, processes, and contexts of refugee mental health and integration are crucial to a deeper understanding of the complexities and nuances involved. The burden of mental health problems and integration continues to be a major concern for refugee groups and policymakers in Norway; therefore, a comprehensive approach to researching refugee mental health and integration in Norway is necessary. Even though the protective and official status that the term refugees entail, and following an inclusivist view (Carling, et al. 2014; 2017), refugees are included as a particular group of migrants in this study.

### 1.1. Successful integration

*Integration* is a concept embedded in different perspectives of the migration process. Research has indicated that migration might be associated with positive mental health outcomes (e.g., sense of belonging) (Beiser and Hou, 2017; van der Boor et al., 2020). However, migrants, including refugees, are more likely to experience reduced social support, decreased life satisfaction, and difficulty accessing necessary services than the major population (Thapa et al., 2007a; Ziersch and Due, 2018; Walther et al., 2020). Refugees, as migrants, invariably undergo adaptation upon resettlement in a new society. Acculturation describes this process of transition and adaptation, referring to the “cultural and psychological change resulting from contact between cultural groups and their members” (Berry, 2003, p. 27). Acculturative stress emerges from conflicts when individuals must adjust to a new culture of the host society and can involve feelings of uncertainty and mental health challenges (Berry, 2021). The process of acculturation may alter the psychological (e.g., behaviors), cultural (e.g., language or religion), social (e.g., ethnic discrimination), and biological (e.g., resistance to diseases) circumstances (Graves, 1967; Berry, 2021) of different migrant groups. Berry’s (2017) model of acculturation suggests four strategies: *Integration* (interest in maintaining both the original culture and from the larger society), *Assimilation* (when individuals do not wish to maintain their cultural identity and seek daily interaction with the larger society), *Separation* (avoid interaction with others and hold onto the original culture), and *Marginalization* (neither interest in own’s culture nor the host society) (23). Each acculturation strategy can mitigate and/or mediate the relationship between migration and mental health depending on how much the acculturating individual emphasizes heritage and/or the settlement culture (Berry, 1997). Individuals will eventually adjust to new situations and changes, and identifying these changes may be necessary to explore (Bhugra et al., 2021). Hence, exploring Berry’s (2021) integration strategy embodies a theoretical concept that can also be debated but is still grounded in the process of adaptation and psychological distress even though other acculturation strategies may be embedded in the migration process. This study focuses on the relationship between different forms of integration (as antecedents) and psychological distress (as an outcome) among Afghan refugees living in Norway.

A successful outcome in the host societies may involve “doing well” in the sociocultural context (e.g., daily cultural living) and “feeling well” in the psychological aspect (e.g., sense of well-being) (Masgoret and Ward, 2006). In the literature, different integration aspects have been measured in several ways (e.g., Sam and Berry, 1995; Birman and Taylor-Ritzler, 2007; Rudmin, 2009; Harder et al., 2018b). The present study draws on the Immigration Policy Lab’s index (IPL-12/24) developed by Harder et al. (2018a) which measures integration from a multidimensional perspective.

To begin with, Harder et al. (2018b) defined integration as “the degree to which immigrants have the knowledge and capacity to build a successful, fulfilling life in the host society (…) rather than the degree to which they have shed their cultural heritage” (p. 2). The latter part of this definition is consistent with Berry’s (1997) idea of cultural maintenance and the sociological view of assimilation (Gordon, 1961; Zhou, 1997; Alba et al., 2012). As Harder and colleagues explain (2018b), the common goal of measuring successful integration is hindered by differences in methods and definitions of integration (p. 11483). The IPL-12/24 index includes six dimensions: political, psychological, social, navigational, linguistic, and economic (Harder et al., 2018b). These dimensions capture different aspects relevant for strengthening the capacity of migrants to establishing a fulfilled life in the new society. With the aid of these dimensions, several studies have been conducted regarding inclusion, assessing the level of integration and help-seeking behaviors among migrants, including refugees (Harder et al., 2018b; Kunwar, 2020; Harris et al., 2021; Passani et al., 2022). To our knowledge, studies using these measures of integration in relation to psychological distress among refugee groups have not yet been conducted in Norway, and hence the focus on the present study.

Migrants, including refugees, are in daily interactions with members of the larger society (Lansford, 2011). These interactions form the basis for integration into the host society. Integration aspects among migrants can be assessed by identifying cultural and socio-economic factors and their behavior and relationship with the host country, for example by addressing basic needs, education, and finding employment (Sheikh and Anderson, 2018; Joyce, 2019; Brell et al., 2020; Stempel and Alemi, 2020). For instance, social integration (Liamputtong and Kurban, 2018; Brydsten et al., 2019; Ali et al., 2021) of migrants in Norway has been associated with good mental health (Dalgard and Thapa, 2007; Thapa et al., 2007b). Among different migrant groups, Harder et al. (2018b) have tried to capture the social aspect (e.g., daily interactions, social ties, feelings of connectedness, and sense of belonging) from both the psychological and the social dimensions of integration in the IPL-24. Other integration indicators include how migrants, including refugee groups, adapt and navigate the host society system (e.g., language use and finding basic services). Integration into the host society includes access to various types of services, such as appropriate use of medical assistance, as Harder et al. (2018b).

### 1.2. Afghans living in Norway

Most Afghans in Norway came either as refugees or asylum seekers (SSB, 2020; UNHCR, 2022). For this study, we focused specifically on the Afghan population living in Norway. According to the Norwegian Living Conditions Report (2018) on the situation of migrants in Norway, 16 % reported mental health problems. This number might be lower than the actual prevalence. Due to political status (e.g., refugees and asylum seekers) in the migration process, some refugees may be reluctant to discuss mental health problems with health professionals based on worries that might impact residence permits (Paniagua and Cuéllar, 2000; American Psychiatric Association, 2013). Distinctly, among the migrant groups, Afghan refugees reported more significant psychological distress and living conditions pitfalls and more mental health problems than other migrant groups (Jakobsen et al., 2017; Straiton et al., 2017; Barstad, 2018; Tronstad et al., 2018). Afghanistan has endured conflicts and wars for many years, and many Afghan refugees have settled in different countries, carrying traumas and mental health problems (Alemi et al., 2017; Slewa-Younan et al., 2017; Ahmad et al., 2019; Mobashery et al., 2020). Afghanistan is among the countries with the highest refugee and asylum seeker status in Norway (UNHCR, 2022), and the changed political situation in Afghanistan since 2022 might further increase the number. Consequently, Afghans fleeing to other countries for safety will have a harder time returning home, increasing the number of asylum seekers and refugees and the mental health burden.

Reportedly, three of four Afghans who came to Norway as refugees and 25 % that has arrived through family reunification, were over-represented in single households and were younger than other migrant groups (Tronstad et al., 2018, p. 30). The mental health problems of Afghan refugees, particularly among younger people, may go untreated, mainly due to the underutilization of mental health services (Anstiss and Ziaian, 2010; Satinsky et al., 2019). The employment rate among Afghans in Norway in 2018 was 62% (higher in the 25–44 age group) compared to other migrant groups (3.1%) (Tronstad et al., 2018, p. 30). Even with a high employment rate, Afghan refugees may still struggle to access mental health services, highlighting the need for further research and outreach. There are also gaps between the dominant and non-dominant groups regarding higher education, for example, household income and language skills (Tronstad et al., 2018). A study of mental public health outcomes found that rates of depression were higher five years after resettlement when living in poor socio-economic conditions (Priebe et al., 2016). Recognizing these disparities highlights the importance of better support systems and resources for refugee groups to integrate successfully into their new communities.

To our knowledge, this is the first study to examine how different dimensions of integration are related to psychological distress among Afghans in Norway. Based on Harder et al.’ (2018b) definition of successful integration, we hypothesize that psychological distress may be associated with all the six aspects of integration suggested by Harder and colleagues. Overall, the different dimensions of integration will be negatively related to psychological distress, i.e., the higher the integration, the lower the psychological distress. Not being nor feeling integrated into the host society may contribute to poor mental health.

## 2. Method

### 2.1. Participant characteristics

With a cross-sectional study design, the current study was part of a broader survey for refugee groups residing in Norway. One-hundred-and-fourteen participants from Afghanistan took part in this study with ages ranging from 18 to 70 years (36% females) with a mean age of 0.36 (SD = 0.482). Over half (53%) of the participants were between the ages of 20 and 29 years. A more detailed socio-demographic description is presented in Table 1.

**Table 1:** Sociodemographic characteristics of the population sample.

Sociodemographic Characteristics	n	%
Gender	0.36 (0.482)	
Males	73	64%
Females	41	36%
Age^a^	2.61 (0.956)	
Age upon arrival in Norway^b^	3.64 (1.023)	
Level of education^c^	0.96 (0.854)	
Have not completed any education	4	3.7%
Primary school	17	15.6%
Secondary school	35	32.1%
Secondary or university education	53	48.6%
Current life situation^d^	2.45 (0.938)	
Unemployed (actively/not actively looking for a job)	19	17%
In school	40	35.7%
In paid work	37	33%
Other	16	14.3%

*N* = 114.

a Age was collected in ten-year brackets (1–7). Min.1 – Max.6.

b Age upon arrival was collected in ten-year brackets (1–7) Min.1 – Max.6.

c Level of education was collected in different alternatives recoded in 4 items. Min.1 – Max.4. Missing *n* = 5.

d Other (Permanently sick or disabled, retired, doing unpaid work, looking after children or other persons). Missing *n* = 2.

### 2.2. Sampling procedure

The study was reviewed and approved by the Norwegian Center for Research Data (NSD notification form: 602214). We included a convenience sample of Afghan adults residing in Norway. Data collection was conducted in two waves: the first wave in the fall of 2019 (September through December), and the 2nd wave in the winter and spring of 2021 (December through April). Data collection was paused during the spring, summer, and fall of 2020 due to the various restrictions put in place at the peak of the Covid-19 pandemic. Most participants (66%) were recruited during the second wave of the data collection (i.e., during the winter and spring of 2021).

A total of 492 individuals opened the survey link. Of these, 271 (55.1%) consented to participate. However, a number of participants did not answer the compulsory questions (e.g., age, age upon arrival in Norway, and gender), and did not proceed further. Participants born in Norway were excluded. A total of 114 (42%) responded to the HSCL-25 scale (i.e., psychological distress) and 112 (41,3%) to the IPL12/24 scale (i.e., integration). There were no missing data points in these scales.

Based on our sample size, we did a sensitivity power analysis (G*Power 3.1.9.6) for multiple regression with 8 predictors to detect a small effect for the selected scales (t-tests for linear multiple regression). A sample of 114 participants would be sensitive to effects of above Cohen’s d = 0.07 with 80% power (alpha = 0.05, two-tailed) (Faul et al., 2009; Bartlett, 2022).

The link to the survey was disseminated *via* flyers and e-mail invitations through different institutions in Norway working with refugees, especially in larger cities of Norway. We also distributed the survey through social media platforms using Facebook ads with categories following standard considerations^1^ to increase participation in the second wave. Informed consent was obtained by all survey participants by ticking off a consent form at the start of the survey. Participants in the survey were not compensated but could participate in a voluntary lottery with the possibility of winning a gift card (NOK500). Participants were also informed that they could contact their general practitioner and municipality refugee health team if they needed to talk to someone after participating in the survey.

The data collected included Norwegian, Dari, and Pashto language versions of the questionnaires. All scales used in this study were translated to Norwegian (except for the HSCL which was available in Norwegian), and the languages of Afghanistan (Dari and Pashto) by a professional translation agency. Translations were revised and back translated by the research team and a research assistant from Afghanistan. The survey was pilot tested prior to the launch of the survey.

### 2.3. Measures

#### 2.3.1. Psychological distress

Depression and anxiety were assessed with the Hopkins Symptoms Checklist (HSCL-25) (Derogatis et al., 1974). The HSCL-25 consists of 10 items about anxiety symptoms and 15 items for depression and somatic symptoms. Each item is rated from *not at all* (1) to *extremely* (4). Cronbach’s alpha for the entire HSCL-25 was 0.95 (0.91 for the anxiety subscale and 0.94 for depression and somatic symptoms subscales). The Hopkins symptoms checklist has been previously used in refugee and asylum seeker populations, including Afghans (Strand et al., 2003; Ventevogel et al., 2007; Jakobsen et al., 2011).

#### 2.3.2. Dimensions of integration

##### 2.3.2.1. Immigration policy lab

The integration questionnaire, Immigration Policy Lab index (IPL-12/24) (2018a; 2018b), assesses six aspects of integration: psychological, linguistic, economic, social, navigational, and political. The scale used in the present study included 20 items from 5 of these IPL-12/24 dimensions. The political dimension of integration was considered less relevant to the aim of the study, and therefore these questions were not included. The dimensions of integration for the current study consisted of interpersonal factors, awareness of general proprieties, and the ability to handle basic requirements in Norway, among others (Harder et al., 2018a). The IPL-12/24 has been validated in international studies (Harder et al., 2018b, p. 11483). In the present study, Cronbach alpha coefficient for the economic (0.62) and navigational (0.35) dimensions were low. Therefore, only single items from these scales were included for the analysis, together with the psychological, social, and linguistical dimensions of integration. The latter dimensions, respectively, had Cronbach alpha 0.72, 0.77, and 0.87. More information about these dimensions follows below.

##### 2.3.2.2. Psychological integration

The psychological dimension included four items that tried to capture the respondents’ feelings of connection with Norway, their wish to continue living there, and their sense of belonging in the host country (e.g., “How connected do you feel with Norway?,” scoring from *I do not feel a connection at all* (1) to *I feel an extremely close connection* (5)).

##### 2.3.2.3. Social integration

This dimension sought to capture the participants’ social capital, social interactions, and social ties. Three items from the IPL-24 were included in the analysis that targeted the frequency of social interaction (e.g., “In the last 12 months, how often did you eat dinner with Norwegians who are not part of your family?”). From *never* (1) to *almost every day* (5).

##### 2.3.2.4. Linguistic integration

The linguistic dimension of integration questions included four components of English communication (i.e., reading, listening, writing, and speaking). The Norwegian survey only used two of these items capturing the ability of reading and speaking the Norwegian language. The two items were: “I can read and understand the main points in simple newspaper articles on familiar subjects,” and “In a conversation, I can speak about familiar topics and express personal opinions.” From *very well* (5) to *not well at all* (1).

##### 2.3.2.5. Selected single items

The economic integration questions captured measures of household income and the ability to meet different levels of unexpected expenses. For the navigational dimension, the measures tried to capture the ability to manage basic needs. Due to the low Cronbach alpha (below 0.60) for both dimensions, we selected single items to represent these dimensions (e.g., “What is your household total annual income?” and “What is your current household? (with estimate alternatives) for the economic; and “How difficult or easy would it be for you to search for a job and see a doctor?,” scoring from *very difficult* (1) to *very easy* (5) for the navigational).

##### 2.3.2.6. Socio-demographic factors

Socio-demographic factors included age, gender, age upon arrival in Norway, current life situation, and educational level. The descriptive statistics showed a positive skew, and the distribution was clustered in the center (e.g., more younger adults answered the survey). The economic dimension of integration also measured a description for *current life situation* referring to the participants’ status in Norway (e.g., unemployed, in school or in paid work). This item was included in the socio-demographic characteristics and not selected for the regression analysis (see Descriptives in Table 1). Questions regarding their political status (e.g., refuge or asylum seeker) were excluded from the survey.

##### 2.3.2.7. Statistical analysis

The online survey was created using the SurveyXact online survey platform.^2^ A report and data set were exported from the SurveyXact to SPSS statistical software program (IBM Corp, 2020). We started with the preliminary analysis and examining the intercorrelations between all study variables. The study relied on the item-total score correlations in evaluating individual responses to the HSCL-25, based on the full 25 items instead of subscales (Lhewa et al., 2007; Lee et al., 2008; Vindbjerg et al., 2021). Next, hierarchical regression analysis was conducted to examine the ability of the different integration variables in predicting psychological distress (measured by HSCL), after controlling for age and education. Due to the lack of significance, gender was not included in the regression analysis. Scores on psychological, social, and linguistic integration and the single items of the navigational and economic scales were included as predictors in the analysis. We also calculated 95% confidence intervals for the regression coefficients based on robust standard errors (see Table 2).

**Table 2:** Hierarchical regression results for psychological distress.

Variable	B	95%CI for B		SE B	*β*	*R* ^2^	△R^2^
		LL	UL				
Step 1						0.032	0.032
Constant	66.7^***^	51.1	82.5	7.91			
Age	−0.925	−4.33	2.47	1.71	−0.05		
Education	−3.46	−7.27	0.344	1.92	−0.17		
Step 2						0.214	0.182
Constant	10.7	−22.5	43.8	16.7			
Age	−0.181	−3.53	3.17	1.69	−0.01		
Education	−1.65	−5.83	2.52	2.10	−0.08		
Household income^a^	−0.51	−2.30	1.29	0.907	−0.06		
Current household^a^	−1.63	−4.35	1.09	1.37	−0.11		
Navigational	2.62	0.70	4.53	0.97	0.28^***^		
Psychological	9.56	−3.14	15.9	3.23	0.27^**^		
Social	2.83	−3.56	9.22	3.22	−0.08		
Linguistic	1.23	−2.27	4.74	1.77	−0.07		

CL, Confidence Interval; LL, lower limit; UL, upper limit. The model in the second step is significant (*p* = 0.002) (*F* (106,100) = 3.856, *p* < 0.0005). ^*^p < 0.05. ^**^p < 0.01. ^***^p < 0.001.

a Single items from the Economic dimension of integration = household income and current household; and Navigational = “How difficult or easy would it be to see a doctor” and “How difficult or easy would it be to search for a job.”

## 3. Results

### 3.1. Preliminary analysis

#### 3.1.1. Socio-demographic variables

Demographic statistics are presented in Table 1. The relationships between psychological distress (HSCL-25) and the socio-demographic variables (IPL-12/24) were investigated using Pearson correlation coefficients. Our analysis ensured no violation of the normality assumptions. Age and education correlated negatively with psychological distress. An independent samples t-test was conducted to compare the psychological distress scores between males and females. The result showed no significant difference in score for males (M = 52.90, SD = 16.2) and females, *M* = 53.46, SD = 18.8; t (112) = −1.66, *p* = 0.86 (two-tailed).

#### 3.1.2. Psychological distress and integration

Both the psychological, the social dimension and the single item of the navigational dimension of integration correlated positively with psychological distress (*r* = 0.269; *r* = 0.049; *r* = 0.358, respectively). Conversely, the linguistic and the economic dimensions of integration showed negative correlations (*r* = −0.067; *r* = −325 and *r* = −0.727, respectively), *N* = 114, *p* < 0.001 (see Table 3).

**Table 3:** Descriptive statistics and correlations for study variables.

Variable	*n*	*M*	SD	1	2	3	4	5	6	7	8	9	10
1. Psychological distress HSCL	114	53.1	17.153	–	0.485ᵃ	0.060	−0.193^*^	0.269^**^	0.049	−0.067	0.358^**^	−0.152	−0.166
2. Gender^a^	114	0.36	0.482	0.485		–							
3. Age^b^	114	2.61	0.956	−0.049									
4. Education^c^	109	0.96	0.854	−0.172	0.019	0.065	–						
5. Psychological integration^d^	113	2.61	0.477	0.269^**^	−0.152	−0.047	−0.006	–					
6. Social integration	112	2.59	0.48426	0.049	−0.078	−0.117	0.165	−0.042	–				
7. Linguistic integration	110	3.97	1.016	−0.067	−0.200^*^	−0.138	0.409^**^	−0.017	0.135	–			
8. Navigational	113	5.91	1.835	0.358^**^	0.006	0.056	0.429^**^	0.064	−0.045	0.250^**^	–		
9. Economic Household^e^ income	112	3.16	1.924	0.325^*^	−0.038	0.206^*^	0.186	0.048	0.080	0.310^**^	−0.305^**^	–	
10. Economic Currently household^e^	112	1.69	1.201	0.727^*^	−0.124	−0.118	0.065	0.086	0.070	0.218*	−0.284^**^		–

^**^p < 0.01. ^*^p < 0.05. Correlation is significant at the 0.01 level (2 -tailed). Pearson product–moment correlations between measures of dimensions of integration and psychological distress (HSCL). A Kruskal-Wallis Test revealed a statistically significant difference psychological distress (HSCL) among the different income levels in household income from the economic dimension of integration (Gp1, Min. *n* = 27: 100000NOK, Gp8 Max. *n* = 3: 1250000), $x^{2}(n=114)=13.96,p=0.052$. In addition, a Mann–Whitney *U* test revealed significance difference in the household income levels (*U* = 10, *z* = −2.111, *p* = 0.035, *r* = −0.02). No significance difference for current household in the economic dimension *U* = 247, *z* = −0.114, *p* = 0.90, *r* = −0.01.

a Females higher than males *r* = 0.016. The magnitude of the difference in the means (mean difference = −0.55, 95%CI: −7.2 to 6.2) was very small (eta squared = 0.002).

b Age was collected in ten-year brackets (1–7). Min. 1 – Max. 6.

c Level of education was collected in different alternatives recoded in four items. Min.1 – Max. 4. Missing *n* = 5 (4.4%).

d Items for the psychological dimension of integration were reversed.

e Single items for the economic dimension of integration (1) Household income collected in 1–8 income brackets Min. 1 – Max 8. Kruskal-Wallis Test (0.325^*^p = 0.052) and (2) Mann–Whitney *U* test (0.727^*^ *p* = 0.035) results when correlated with HSCL.

Hierarchical multiple regression was performed to assess the ability of the dimensions of integration to predict psychological distress (HSCL) after controlling for the sociodemographic variables (age and education). Preliminary analyses were conducted to ensure no violation of the assumptions of normality, linearity, multicollinearity, and homoscedasticity. Age and education were entered at Step 1, explaining 3% of the variance in psychological distress. After entry of the single predictors of the economic and navigational dimensions and the three dimensions of integration (psychological, social, and linguistic) in Step 2, the total variance explained by the model as a whole was 21.2%, F (9,99) = 2.96, *p* < 0.004. Thus, the predictors explained an additional 18% of the variance in psychological distress after controlling for age and education (R squared change = 0.18, F change (6,99) = 3.762, *p* < 0.001). In the final model, only two predictors were significant, the single item of the navigational dimension (i.e., How difficult or easy would it be for you to search for a job and see a doctor?) (beta = 0.28 *p* < 0.010) and the psychological dimension (beta = 0.27 *p* < 0.004) (see Table 2).

## 4. Discussion

This study explored how different aspects of integration are related to psychological distress among Afghan refugees living in Norway. Following Harder et al. (2018a), we examined whether the level of psychological distress (assessed with HSCL-25) might be associated with various aspects of integration. Our conceptualization allowed us to assume that psychological distress might be a function of different aspects of integration rather than focusing on integration as a unitary global concept.

Our analysis revealed that only the psychological dimension of integration contributed significantly to psychological distress. However, the portion of explained variance was modest. Neither the social nor the linguistic dimensions made a significant contribution in explaining psychological distress. These dimensions had low correlation with psychological distress. The single item used to measure the navigational dimension of integration significantly contributed to explain psychological distress. The results suggest that the psychological dimension of integration seems to be a better predictor of psychological distress with a favorable outcome among Afghans. Our findings align with previous research showing that feelings of connection with the new country are important for mental health outcomes (Dalgard and Thapa, 2007; Kashyap et al., 2019; Strømme et al., 2020). Thus, our results suggest that the risk for psychological distress might be reduced when refugees feel connected to the Norwegian society.

There were moderate inter-correlations between most of the integration scales. Feeling connected and not isolated from the Norwegian society may enhance other aspects of integration, such as contacting the local community, learning how to navigate the system, and seeking basic needs (Harder et al., 2018b). Although the direction of effects cannot be established by correlations, it is possible that the psychological dimension of integration could act as a facilitator for successful integration. Within the psychological dimension, refugees who feel safe in the host country can self-regulate and gain agency, contributing to successful integration, also in the other dimensions of integration (Harder et al., 2018a,b). On the other hand, other aspects of integration, such as language skills, may also facilitate the psychological connectedness, for example, by giving motivation for employment and social network.

In this study, a positive psychological dimension of integration may reverse the outcome of psychological distress if one never feels isolated or as an outsider in Norway. In the adaptation stage the affective and emotional issues are often the last to be addressed when accepting and acknowledging cross-cultural differences (Bhawuk et al., 2006). As a result, integration may go beyond cross-cultural tolerance, and individuals may become multicultural in their way of thinking and being (Bhawuk et al., 2006; Huynh et al., 2011; Landis and Bhawuk, 2020). The adaptation process is characterized by empathy and cultural diversity leading towards integration. For example, Afghans may reach out to others from their own country or turn to other migrant groups for social support when they find themselves in similar situations or need help navigating the system instead of or in addition to their Norwegian counterparts.

The non-significant effects for the linguistic dimension of integration in our study do not necessarily indicate lower proficiency in the Norwegian language nor that Afghans are less likely to identify with the Norwegian culture. However, not being able to speak the language and communicate with others could be somewhat of a burden that can hinder everyday interactions (e.g., not knowing how to meet basic needs nor accessing services can lead to feelings of isolation that can worsen refugees’ mental health). Feeling safe and connected to the Norwegian society, on the other hand, might help refugees develop the motivation to learn the language and search for work. The significant (negative) contribution of the individual element of navigation on psychological distress suggest that knowing how, when, and where to seek help might be relevant for mental health; despite certain barriers (e.g., cultural traditions, stigma, discrimination) that may prevent Afghans to navigate the system. Previous studies on integration have been supported in aspects related to these dimensions (e.g., Dalgard and Thapa, 2007; Thapa et al., 2007a; Straiton and Myhre, 2017; Walther, 2021).

When comparing the dimensions of integration (Harder et al., 2018b), we found that despite the non-significant results from some of the predictors, reaching a certain level of integration with, for example, the psychological dimension can be considered a sign of successful integration. Connecting with Norwegian society, regularly speaking the Norwegian language, getting along in the community, and seldom feeling like an outsider strengthens the integration process and the individual’s ability to act. These references are consistent with several core integration areas identified previously, such as employment, education, social connections, rights acquisition, language, cultural knowledge, and stability as facilitators (e.g., Ager and Strang, 2008). Nonetheless, inaction in mental health situations (e.g., refusing treatment or not accepting a diagnosis) could affect a person’s ability to navigate the system, leading to for example, the underutilization of health services. In addition, good health status, reliable access to health care, and support for health outcomes were widely viewed as essential resources for active engagement in the new society (Ager and Strang, 2008). Local channels could also mitigate several factors by promoting mental health information (e.g., formal and informal information in different languages) or facilitating integration with behavioral training (community services from health institutions or voluntary organizations) (Bhawuk et al., 2006; Straiton and Myhre, 2017; Tschirhart et al., 2019).

Social ties between Afghans and language barriers may not interrupt the adaptation process, especially if collective feelings of attachment to Norway are positively involved (de Smet et al., 2019; Frounfelker et al., 2020). Some experiences may include feelings of security and social support from family, friends, and the Norwegian society, contributing positively to the integration process. These experiences have been confirmed in previous research through focus group interviews with some refugee groups in Norway (Markova and Sandal, 2016; Aarethun et al., 2021; Brea Larios et al., 2022). Some intercultural training on refugee integration could be conducted to improve successful integration (Bhawuk et al., 2006; Landis and Bhawuk, 2020). In reports on different generations and migrant groups, we could find positive results (Brekke et al., 2021; IMDi, 2021; Bhawuk, Landis & Lo in Sam and Berry, 2006; Strømme et al., 2020), and often socioeconomic factors among other indicators were related to the psychological well-being (of resettled refugees) (Li et al., 2016). Not feeling isolated from Norway can provide some relief from a previous traumatic event.

The associations between psychological distress and integration have important implications for future prevention and integration efforts. The particular significance of the psychological dimension and the single navigational item compared with the non-significant results, suggest other mechanisms for successful integration. While our study suggests that only the psychological dimension is related to psychological distress this result points to potential benefits of measures facilitating inclusiveness. For instance, training of migrants, including refugees, emphasizes behavioral outcomes such as affective and emotional aspects, and cross-cultural tolerance from both dominant and non-dominant groups as part of the adaptation process (Bhawuk et al., 2006; Doucerain, 2019; Landis and Bhawuk, 2020).

## 5. Limitations

The present study had some limitations. First, the psychological distress measure is not a diagnostic tool, nor does it distinguish between different mental health conditions in refugee groups. This study focused on Afghans as a national group living in Norway, and the native population, i.e., Norwegians were not used as a point of reference to compare success in Norwegian society. Even though results have been interpreted with caution, causal conclusions are not drawn from these results from the cross-sectional study. In contrast to the results of this present study, previous research has indicated significant effects on, for example, on social integration (Dalgard and Thapa, 2007; Abebe et al., 2014).

Our study included five dimensions of integration, however, only three dimensions (psychological, social, linguistic) and two single items drawn from the economic and navigational dimensions (due to the low internal consistency and low Cronbach’s alpha), were included in the analysis. We, performed a classic multiple linear regression analysis, and the test results confirmed the findings with some of the claims supported by the data. The IPL team has previously done studies among larger immigrant groups in the United States; other dimensions in this study could have been collected with a larger group of Afghan participants with refugee backgrounds or from other refugee groups in Norway (Harder et al., 2018a,b). Future studies should replicate our findings on a larger and more heterogeneous sample.

There were more males than females, and the majority were relatively young. Participation in the survey could have been restricted due to difficulties accessing and completing the survey. Most of our participants were young adult males, and lower participation in the survey may be associated with difficulties in accessing and completing the survey (e.g., lower education (non-literacy) among potential participants or lower social media use among females), which may not help achieve the required number of participants. Whether the findings suggested that it holds for younger or male participants than for females, responses hold a relevant case for this study. The questions applied to all Afghans living in Norway (in terms of the categories selected for the Facebook ads and inclusion criteria relevant to the Afghan population), and the survey ruled out questions directed specifically to the respondents (e.g., information on refugee status). Anonymity and participant protection played an important role.

The study also had difficulties collecting sufficient data and recruiting participants. Interpretation bias may affect future predictions and decisions in psychology research; however, caution is warranted when interpreting data from small samples (Hekler et al., 2019). During the first wave of covid and after the pandemic breach, data collection may have affected the responses. However, despite the low response rate before the pandemic, some questions could influence the answers – in this case, questions from the social and linguistic dimensions of integration – given the non-significant contribution. Nevertheless, unforeseen circumstances may have required some adjustments to data collection, such as cluster sampling. Furthermore, people with lower digital literacy might have been excluded from a self-administered online survey, reducing the likelihood of participation among those with lower education levels and language proficiency. The results of our study may underestimate the non-significant effects and may not favor those who feel connected to the Norwegian society over those who integrate with the other dimensions of integration. For example, displacement time (number of years) could be considered when analyzing the different aspects of successful integration. In the survey, this question was not included. Instead, only the age at which subjects arrived in Norway was collected in ten-year brackets. The factor aspect can also be considered through other statistical analysis. Nevertheless, the data has yielded valuable responses and contributed to the research study. For a deeper understanding of pre- and post-migration determinants and identify effective strategies to improve the mental health and well-being of refugees, further research could consider the target population, the duration of stay in Norway, the setting and time of analysis, and the pandemic situation.

## 6. Conclusion

*Integration* gathers different perspectives of the migration process. The current study has shown that the psychological dimension of integration can predict psychological distress affecting the Afghans’ adaptation process in Norway towards successful integration. After the migration process, the relationship between integration and psychological distress could influence the path to successful integration, with the psychological dimension contributing to the sense of belonging in Norway. Afghans can adapt better and improve their mental health if they feel connected and have a sense of belonging in the Norwegian society. The different understanding of integration goes hand in hand with the different dynamics presented. However, using only one integration dimension to explain successful integration may pose certain issues for refugee integration in the Norwegian society and integration policies. In refugee settlement, integration is a central concept that plays out in different aspects in different contexts. This could be addressed in cross-cultural trainings to further explore and develop integration appropriate for the migration process. Despite this, integration is still measured in labor market activities, educational attainment, and social environments in the community. After the rapid withdrawal of U.S. and NATO forces from Afghanistan at the end of 2021, a new wave of Afghan refugees could emerge. The situation in Ukraine also challenges the Norwegian society and other immigrant groups with another wave of refugees. A balanced discussion of integration policy and advanced mental health training are crucial (e.g., interventions and prevention options to reduce symptoms of mental health problems in Norwegian health care facilities - from screening to referral in centers and clinics when screening is positive).

Mental health care services are often difficult to access for refugees. Providing information about entitlements and available services, facilitating social integration, developing outreach programs, and training professionals to work with these groups are all best practices for developing effective mental health care. Integration plays a vital role in all forms of migration, but refugee groups face most challenges despite current support programs. It is essential to develop programs that consider interactions and opportunity structures to understand the integration process better. Refugee integration should expand beyond focusing on homogeneity and heterogeneity to influence and build structures that shape refugee integration. The different dimensions of integration are a starting point to help develop more information for future migration policy.

## Data availability statement

The raw data supporting the conclusions of this article will be made available from the authors, without undue reservation.

## Ethics statement

The studies involving human participants were reviewed and approved by Ethics committee/IRN of the Norwegian Center for Research Data (NSD) Notification form: 602214. The participants provided their written informed consent to participate in this study.

## Author contributions

DBL, DS, and GS contributed to the revision and writing of the manuscript and had primary responsibilities for the data analysis. DBL created the tables. All authors contributed to the article and approved the submitted version.

## Funding

This study is part of a larger project aimed at building a research-based platform to improve mental health services and the needs of refugees in Norway. Our work was funded by the Research Council of Norway (Project number 273645).

## Conflict of interest

The authors declare that the research was conducted in the absence of any commercial or financial relationships that could be construed as a potential conflict of interest.

## Publisher’s note

All claims expressed in this article are solely those of the authors and do not necessarily represent those of their affiliated organizations, or those of the publisher, the editors and the reviewers. Any product that may be evaluated in this article, or claim that may be made by its manufacturer, is not guaranteed or endorsed by the publisher.

## Footnotes

^1^ https://www.facebook.com/business/ads

^2^ www.surveyxact.no

## Abbreviations

HSCL-25: Hopkins symptoms checklist
IPL-12/24: Immigration policy lab
PTSD: Post-traumatic stress disorder
